# Aging Processes Are Affected by Energy Balance: Focused on the Effects of Nutrition and Physical Activity on Telomere Length

**DOI:** 10.1007/s13668-024-00529-9

**Published:** 2024-03-18

**Authors:** Satı Gürel, Elif Nisa Pak, Nilüfer Acar Tek

**Affiliations:** 1 Department of Nutrition and Dietetics, Faculty of Health Science, Trakya University, 22030 Edirne, Turkey; 2 Department of Nutrition and Dietetics, Faculty of Health Science, Kilis 7 Aralık University, 79000 Kilis, Turkey; 3 Department of Nutrition and Dietetics, Faculty of Health Sciences, Gazi University, 06490 Ankara, Turkey

**Keywords:** Telomere length, Nutrition, Energy balance, Physical activity, Aging

## Abstract

**Purpose of Review:**

The number and proportion of individuals aged 60 and over are increasing globally. The increase in the elderly population has important social and economic effects. Telomere length is an important marker for healthy aging. Here, we review the relevance between telomere length and energy balance by determining the effects of physical activity, nutrients, dietary patterns, and foods on healthy aging and telomere length with related studies.

**Recent Findings:**

Evidence emphasizes the importance of telomere length and integrity for healthy aging. It also focuses on the importance of potential interventions such as physical activity and a healthy diet to improve this process.

**Summary:**

We suggest that ensuring energy balance with regular physical activity and healthy diets can contribute to the aging process by protecting telomere length. In addition, different methods in studies, short and inconsistent durations, different types of exercise, different diet patterns, and non-standard foods have led to conflicting results. More studies are needed to elucidate molecular-based mechanisms.

## Introduction

The number and proportion of individuals aged 60 and over are increasing globally. It is stated by the World Health Organization that the elderly population, which was 1 billion in 2019, may double in 2050 [1]. This increase will accelerate in the coming decades. The increase in the elderly population has important social and economic effects. Aging brings with it a progressive loss of physiological integrity, and this is a risk factor for chronic non-communicable diseases [1]. Maintaining healthy behaviors, especially a balanced diet and regular physical activity throughout life, contributes to reducing the risk of non-communicable diseases, improving physical and mental capacity, and healthy aging [2]. Today, strategies to prevent age-related adverse events are an important public health intervention [3]. Research on aging has advanced in the last years, with the discovery that the rate of aging is controlled by biochemical processes and genetic pathways such as mitochondrial dysfunction, cellular senescence, stem cells, and telomere attrition [4]. Recent findings have focused on the importance of telomere length and maintenance for healthy aging, as well as the importance of potential interventions to the stature of this process, such as a healthy diet and physical activity [3].

Therefore, the purpose of this review is to survey the relevance between telomere length and energy balance by determining the effects of physical activity, nutrients, dietary patterns, and foods on healthy aging and telomere length with related studies.

The study is poised to contribute to the burgeoning literature by addressing several noteworthy research gaps. Firstly, the interaction between various lifestyle elements such as physical activity, nutrient intake, dietary patterns, and specific foods remains an understudied terrain. Our review aspires to elucidate the combined effects of these factors, offering a holistic perspective on how they collectively influence telomere length and, consequently, the aging trajectory. Secondly, while previous research acknowledges the link between lifestyle factors and telomere length, a comprehensive understanding of the intricate cellular mechanisms involved in this interaction remains elusive. Our review aims to delve deeper into these molecular pathways, shedding light on the biological underpinnings that mediate the effects of nutrition and physical activity on telomere maintenance. In addition, the study ventures into the realm of individual variability, a facet that has garnered less attention in the existing literature. By encompassing these research gaps, our review article endeavors to contribute to a more nuanced comprehension of the interplay between energy balance, telomere length, and aging processes. In doing so, we aspire to provide valuable insights for both researchers and practitioners, offering a foundation upon which further studies can build to enhance our understanding of healthy aging strategies.

### Telomere Length and Aging

Telomeres are structures consisting of hexanucleotide sequences (TTAGGG) at the ends of eukaryotic chromosomes, containing distal single-stranded and proximal double-stranded regions [5]. TTAGGG sequences that constitute telomeric deoxyribonucleic acid (DNA) form a complex by binding with Shelterin. This complex is one of the important compositions that protect the chromosome ends by shaping the telomere structure [6]. Adult mitotic cells that lack replication of certain terminal segments, such as the ribonucleic acid (RNA) primer, shorten during each cell division [7]. When these shortenings in telomeres reach a critical point, the cell cycle is disrupted, and genomic instabilities and replicative senescence occur [5].

Telomere length, considered a complex inheritable property, is related to aging and age-related diseases. While longer telomeres are associated with a healthy diet, ideal body weight, avoidance of smoking, and physically active life, short telomeres have been associated with metabolic factors such as oxidative stress and increased inflammation [6, 8–10]. Moreover, telomere shortening has been reported to be associated with modifiable lifestyle-related conditions such as abdominal fat, high blood sugar levels, and a sedentary lifestyle [11]. Studies have claimed that telomere length is regarded as a good biomarker of aging, as well as that telomere shortening may be a molecular clock that triggers aging [12]. Telomere length is supposed to be stable from childhood to young adulthood although there are individual differences but begins to decline at older ages [13]. In addition, it was stated that telomere length was positively associated with the number of healthy life years [14]. While the evidence is compelling for links between telomere length and certain age-related metabolic processes, the causal relationship between age-related telomere shortening and aging-related diseases remains unclear. Whether telomere shortening is the cause or merely the result of diseases is still under investigation [7].

## Effects of Physical Activity on Telomere Length

It is known that physical activity positively affects both mental and physical health. Studies have reported that physical activity prevents aging-related diseases, supports healthy aging, and is associated with increased life expectancy [15]. However, physical activity of moderate intensity and exercise have been reported to reduce inflammation and oxidative stress [16].

It is known that telomere length decreases with aging and age-related diseases accelerate this process. Increasing oxidative stress, inflammation, and a decrase in telomerase activity with aging accelerate the shortening process of telomeres [9]. Considering the positive effect of physical activity on healthy aging and that telomere length may be a biomarker of healthy aging, physical activity may be associated with telomere length. A recent meta-analysis that included 30 studies (7418 individuals) has found that physically active participants had longer telomeres than sedentary participants (SMD = 0.70, 95% CI 0.12–1.28, very low certainty) [17••]. Cherkas et al. [18] stated that leukocyte telomere length is 200 nucleotides longer in physically active individuals than in individuals with low physical activity (*P* < 0.001). In the study, it was suggested that this relationship was also significant according to age, and accordingly, regular physical activity could prevent aging. A study induced in elderly women reported that individuals with physical activity levels ≥ 17 MET-hours/week had longer telomere base pairs than individuals with < 1.25 MET-hours/week [19]. In addition, it has been reported that the duration, intensity, and persistence of physical activity are positively related to telomere length [20]. The association of differents sports types across different periods of life on telomere length individuals over 61 years of age form the Berlin study of aging II (BASE-II) was analyzed. According to the study, telomere length was positively correlated with current physical activity. In addition, practicing sports for 10 years or more has a positive effect on telomere length; the highest significant effect was observed in participants who played intense activity sports for at least 42 years [21]. Another study found that a 6-month program of physical exercise increased the relative telomere length in adults over 65 years of age [22]. Ludlow et al. [23] showed that the telomere length of elderly participants with an exercise energy expenditure of 0–990 kcal/week and > 3540 kcal/week was shorter than individuals with an exercise energy expenditure of 991–2340 kcal/week. In a study by National Health and Nutrition Examination Survey (NHANES), intense leisure-time physical activity increases by 1 h per week and an increase in total moderate physical activity by 1 h per week were importantly associated with longer telomeres (respectively 0.31%; 0.08%,) [24]. In Fretts et al. [25] study, participants with more steps per day had longer telomere lengths than participants with fewer steps per day. This may show that ambulatory activity positively affects telomere length. In an NHANES study, the movement-based activity index (MBB) was established. According to the results of the study, a dose–response relationship was shown between MBB involvement and telomere length. Accordingly, the telomere shortening of the elderly, who are more active in daily life, may slow down [26].

Although most of the current studies indicated that physical activity is positively related to telomere length, conflicting results have also been reported. Generally, these inconsistencies are attributed to reasons such as gender, type of exercise, the intensity of physical activity, and the presence of obesity [27•, 28]. Two recent studies reported a favorable relationship between physical activity and telomere length in Jantunen et al. [29] only in female participants and Stenback et al. [30] only in male participants. In an NHANES study of obese individuals, all active individuals, except those who were obese/overweight for a longer period of time, were associated with longer telomeres compared to sedentary individuals. Accordingly, it has been suggested that the presence of obesity for a long time may adversely affect the telomere protective impact of physical activity [31]. Substantial evidence suggests that physical activity is associated with longer telomeres, indicating a potential link to healthy aging. However, discrepancies in findings can be attributed to factors such as gender, exercise type, intensity, and obesity. Further research is needed to clarify these inconsistencies and to better understand the mechanisms underlying the relationship between physical activity and telomere length in the context of aging.

### The Effect of Different Types of Exercise on Telomere Length

The effects of physical activity on telomere length may vary depending on the type of exercise [32]. Werner et al. [33] have compared the long-term effects of three exercise modes (resistance training, endurance training, and interval training). They suggested that telomerase activity and telomere length, which are important for regenerative capacity, cellular aging, and therefore healthy aging, increase with interval training and endurance training, but resistance training does not show the same effect. Balan et al. [34] showed that endurance training positively affects telomere length through TERRA. A similar study was found that long-term endurance exercise promoted the preservation of telomere length in older ages [35]. Rosa et al. [36] studied the effects of sprinting and endurance training on biomarkers of aging in veteran athletes. All participants showed better inflammatory status and redox balance compared to control. By exercise type, sprint training had a better cytokine profile, redox balance, and reduced biomarkers of aging, while a better nitrite/nitrate (NO–) profile was observed for endurance training as a marker of endothelial function. Short-term aerobic exercise training did not affect telomere length in obese women with polycystic ovary symptom [37]. In a study examining the effect of 12-week resistance training on telomere length, it was noted that although no change was observed in telomere length after the intervention, there were improvements in molecular parameters related to telomere integrity [38].

These studies suggest that certain exercise modalities affect cellular aging regulators differently. However, comparative studies are few in the available literature. The diverse and intricate nature of the factors influencing the relationship between physical activity and telomere length underscores the complexity of this association. The disparities in study populations, exercise types, methodologies, and individual responses collectively contribute to the conflicting outcomes observed across the literature. It is evident that a comprehensive comprehension of this intricate relationship necessitates more nuanced and tailored research designs that account for these multifactorial influences.

### Effects of Energy Balance, Weight Loss, and Caloric Restriction on Telomere Length

The relationship between energy metabolism and longevity has been a key topic in aging research for many years [39–41]. One of the theories linking energy metabolism and longevity is the oxidative stress theory of aging. Accordingly, harmful productions of oxidative metabolism (such as oxidative stress and reactive oxygen species) accumulate in cells and cause damage [42]. This damage is associated with cellular aging. In many studies, it was revealed that the level of oxidative damage in macromolecules increases with age, and that genetic interventions and dietary restrictions reduce oxidative damage while extending lifespan [39, 42, 43]. The effects of energy metabolism via telomeres on aging and longevity are not found in studies. However, when the mechanisms of telomere’s impact on aging are examined (also explained), there may be a relationship between energy metabolism and telomere length. In this context, studies that show the relationship between factors related to energy metabolisms, such as weight loss and calorie restriction, with telomere length may support this hypothesis. In a study observing the relationship of weight loss intervention (6 months) to telomere length, weight loss was positively correlated with telomere prolongation. It has been shown that telomere length increases as weight loss increases. Also, the highest effect was seen in individuals with the shortest telomeres at baseline [44]. In a similar study, it was reported that 10% or more weight loss and maintenance can provide telomere elongation in individuals who are intervened with calorie restriction and exercise program for 12 months [9]. In a study conducted on obese men, it was found that weight and body fat loss achieved with calorie-restricted diets resulted in gains in telomere length [45]. There was no change in telomere lengths in obese individuals who lost weight with bariatric surgery [46]. Similarly, it was observed that 12 months of diet and exercise intervention did not change telomere length in post-menopausal women [47]. In the backdown, studies viewing the relationship between energy balance and telomere length have generally been conducted on calorie restriction, reducing energy intake through weight loss, and increasing energy expenditure through physical activity and exercise programs. Studies are limited in number and contain conflicting results. The intricate relationship between energy metabolism and telomere length is influenced by a complex interplay of genetic, environmental, and lifestyle factors. The inconsistent results observed across studies can be attributed to methodological variations, participant heterogeneity, and the multifaceted nature of energy metabolism’s influence on cellular processes. A more comprehensive approach encompassing diverse populations, rigorous methodologies, and a broader spectrum of energy metabolism aspects is essential to elucidate the true nature of this intricate relationship. In addition, there was no study examining the relationship between the basal metabolic rate or resting metabolic rate and telomere length.

### Potential Mechanisms in the Effect of Energy Balance on Telomere Length

**Fig. 1:**
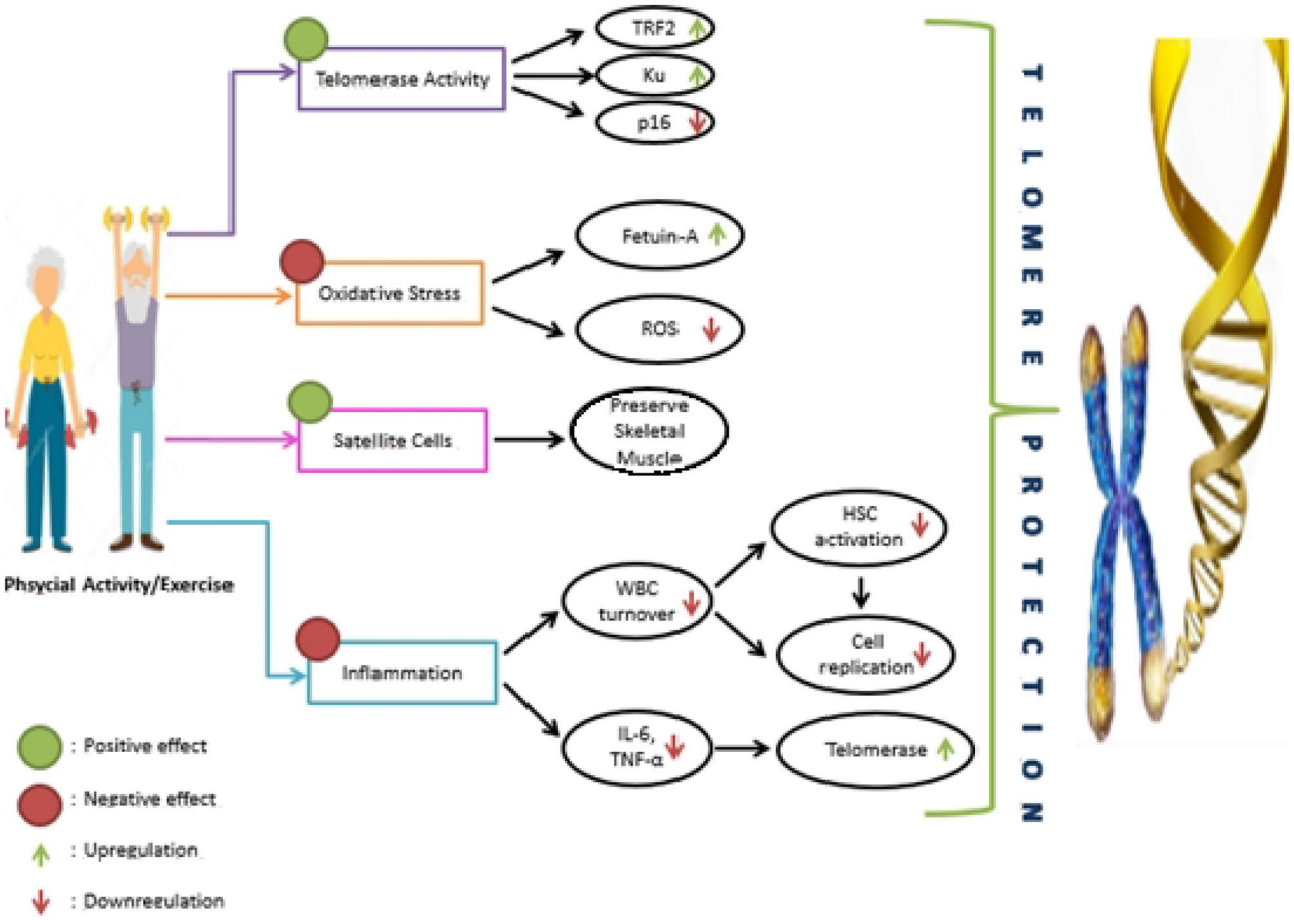
The potential effects of physical activity and exercise on telomere length. HSC, hematopoietic stem cell; TRF2, telomeric repeat-binding factor 2; TNF-α, tumor necrosis factor-α; ROS, reactive oxygen species; IL, interleukin; WBC, white blood cell

The aging process is considered to be associated with decreased telomere length. Generally, the current literature has focused on energy expenditure through physical activity in the energy balance-telomere length relationship [16]. There is no study on the relationship between reducing energy intake and telomere length. Therefore, potential mechanisms in the energy balance-telomere length relationship are explained based on the effect of physical activity on telomere length. Although current studies do not provide sufficient evidence, the baseline mechanisms mentioned are telomerase activity, oxidative stress, skeletal muscle satellite cell content, and inflammation (Fig. 1) [48–50].

#### Telomerase Activity

Telomerase enzyme regulates, maintains telomere length, and defines cellular replicative capacity. Physical activity positively affects telomerase subunit TERT, shelterin component telomeric repeat-binding factor 2 (TRF2), and DNA repair factors KU and p16 proteins (Fig. 1) [51]. Since these molecules are considered important factors for telomerase activity, an enhancement in telomerase activity after exercise may support telomere elongation. In a study comparing athletism athletes and sedentary adults, they found an upregulation of TRF2 in athletes that plays a role in preventing telomere shortening [52]. A study tested telomerase activity after different durations (30, 60, and 90 min) of high-intensity interval cycling exercise in older and younger subjects. Accordingly, TERT messenger RNA (mRNA) levels increased in both groups, but the increase was greater in the young [53]. Chilton et al. [54] reported that exercise has the potential to control the upregulation of TERT mRNA and downregulation of TRF2 mRNA, in addition to and downstream expression of microRNAs (miRNA) involved in telomere homeostasis. These studies confirm that one of the mechanisms by which physical activity protects against aging may be to increase telomerase activity.

However, studies show that the increase in telomerase activity after acute exercise is temporary. In a study, 34 people followed an endurance training program (3–5 times/week in 40-min periods; aerobic exercise), while the other half were sedentary for 24 weeks. As a result of the study, while telomere length was increased in the trained individuals, telomere length was lightly shortened in the sedentary group, and also no change was observed in telomerase activity in either group [55].

With the findings from the studies, the exact kinetics of higher telomerase activity due to physical activity still need to be determined.

#### Oxidative Stress

The rate of telomere shortening is not constant from birth and probably has been varied from a division cycle to another depending on oxidative stress, defense antioxidants, and even cell type [56]. Excessive production of reactive oxygen species (ROS) can cause oxidative stress in cells, tissues, or organs, leading to DNA damage, telomere attrition, and aging [57, 58]. 

It is accepted that moderate and regular physical activity can reduce the effect of aging by reducing the level of oxidative stress [49]. Major mechanisms associated with physical activity and telomeric shortening include reduction of ROS, promotion of the expression response in antioxidant proteins and DNA repair enzymes, and REDOX balance (e.g., levels of fetuin-A) (Fig. 1) [59, 60]. During exercise, ROS production is temporarily increased. This increase is an antioxidant response that promotes existing cellular and molecular pathways that increase the resistance of cells and organisms to subsequent greater stress [3, 61]. In one study, it has been reported that exercise ameliorated the age-related decline in mitochondrial oxidative capacity by activating AMP-activated protein kinase (AMPK) which is the metabolic energy deprivation sensor, and the PGC-1 redox signaling pathway [62, 63]. Another study found a contrary relevance between aerobic capacity and blood oxidative stress biomarkers in older Mexican adults [64].

Studies have shown that physical activity can affect telomeres through the regulation of ROS. However, understanding the causes and boundaries between the “beneficial effects” and “harmful effects” of ROS is one of the most complex issues in physical exercise biology [62]. More studies are needed on exercise-induced ROS signaling and its effect on telomeres.

#### Inflammation

Critically short telomeres trigger aging and eventually cell death. One of the factors affecting this shortening rate is inflammation. This effect is associated with overexpression of circulating inflammatory cytokines such as interleukin-6 (IL-6) and tumor necrosis factor-α (TNF-α). These cytokines can downregulate telomerase, resulting in telomere shortening. In addition, inflammation increases white blood cell (WBC) turnover, increasing the division of hematopoietic stem cells and cellular replication, thus bring to telomere shortening (Fig. 1) [65, 66]. In a study conducted on Cushing’s syndrome (mean age = 48.6 ± 12.8 years), telomere length was inversely related to IL-6 and an inflammation marker C-reactive protein (CRP) [67]. A similar study found a significant and decreasing linear trend in telomere length as men’s CRP levels increased; however, this relationship was not observed in women [27•]. In a study conducted on postpartum women, a significant inverse correlation has been found between telomere length and IL-6 [68]. O’Donovan et al. [69] suggest that the inflammatory burden increased by the combination of high TNF-α and IL-6 levels is associated with ascented rates for short telomere length.

In a recent study, elite athletes who did high-intensity endurance sports had longer telomeres and higher levels of IL-6, TNF-a, and anti-inflammatory cytokines. According to the researchers, this suggests less aging in higher-intensity endurance sports associated with heightened immune response [70]. This study may show that physical activity is protective against the negative effect of inflammation on telomere length.

#### Satellite Cells

Satellite cells are responsible for the growth of muscle fibers and the repair of damaged muscle fibers. These cells are activated by stimulants such as increased muscle tension and muscle fiber injury. [71]. It has been reported that the number of satellite cells decreases after the age of 70, and this may contribute to the decrease in muscle mass seen in sedentary individuals [72]. A study on elderly women reported a positive relationship between the count of satellite cells and skeletal muscle telomere length [52]. Physical activity is a factor stimulating the satellite cell pool that counteracts the increasing loss of muscle mass with aging. Even during normal daily physical activities, satellite cells are constantly being renewed [73]. It has been asserted that the shortening of satellite cell telomeres leads to decreased replication capability in satellite cells [16, 73]. In this context, physical activity may serve to protect telomeres and muscles through satellite cells [72]. Darr and Schultz [74] have found that eccentric exercise increases muscle fiber hypertrophy and stimulates satellite cell activation. A study has determined that exercise resulted in muscle hypertrophy and improved muscle regeneration in mice [75].

Studies indicate that satellite cells are a potential player in the relationship between telomere length and physical activity. However, the mechanism has not been fully elucidated. In particular, more studies on humans are needed.

## Effects of Nutrition on Telomere Length

### The Effect of Dietary Pattern on Telomere Length

The variety and amount of foods consumed affect health and aging. One of the theories of aging is the shortening of telomere length. When telomeres, which shorten with each cell division, fall below a critical limit, cells age, or die. The rate of this shortening of telomeres can also affect the rate of aging. The shortening of telomere length, which varies between individuals, can be affected by genetic factors and environmental factors such as stress and lifestyle. Nutrition, which is one of the most important determinants of lifestyle, can affect the aging mechanisms associated with telomere shortening [76]. When the long-term effects of nutrition on health are analyzed, dietary patterns are at the forefront rather than just a single nutrient in people’s daily diets. Telomere lengths of individuals with different dietary patterns change differently over the long term [76, 77].

The positive effects of the Mediterranean diet model and the Dietary Approaches to Stop Hypertension (the DASH diet) on health have been widely accepted. Both diet models are rich in antioxidant vitamins, minerals, polyphenols, phytochemicals, and dietary fiber [78, 79].

The DASH diet is a recommended diet to prevent prehypertensive patients from developing hypertension [79]. The Mediterranean diet, on the other hand, is a natural nutritional model with many health benefits. Mediterranean diet typically includes plenty of vegetables and fruits, whole grain products, legumes, nuts, and high amounts of fiber, and moderately low-fat dairy products, chicken, fish (2–4 times/week), and limited amounts of red meat (1–2 times /month). Olive oil is the main source of fat for this diet. Red wine may be consumed in moderate amounts (1 glass/day for women, 2 glasses/day for men) [78, 80]. The Mediterranean diet, which is accepted as one of the healthiest diet models globally, has the efficacy of reducing oxidative stress and inflammation markers [81, 82]. Thanks to its rich bioactive compounds, this dietary model has beneficial effects on DNA repair and telomere length markers and promotes healthy aging [82]. In a prospective study conducted by Trichopoulou et al. [83] on 22,043 adult individuals, a decrease in total mortality is positively associated with adherence to a Mediterranean diet. One of the reasons for these positive effects of the Mediterranean diet on telomeres may be the limited red meat in the diet and the consumption of other protein sources at the recommended frequency. It is noted that ROS formation and oxidative stress in mitochondria can be reduced by protein (methionine) restriction in a daily diet, and thus maximum life span can be affected [84]. The relationship between telomere length and diet quality was evaluated in Hispanic subjects using 5 different evidence-based dietary indices (PDQ-Prime diet quality score, FQI-fat quality index, MEDAS-Mediterranean diet compliance screening, AHEI 2010-alternative healthy eating index, and DASH-Dietary approaches index to stop hypertension). It has been shown that the risk of having short telomeres is low in individuals with high diet quality indices [85•]. The prospective cohort study conducted by Crous-Bou et al. [86] on 4676 healthy female nurses also supports this study. Alternative Mediterranean Diet scores were calculated with the data obtained from the food consumption records of the participants. As a result of the analysis, each one-point change in Alternative Mediterranean Diet scores corresponds to an average of 1.5 years of aging in terms of telomere length. As a result, adherence to the Mediterranean diet was positively associated with longer telomeres [86]. In a cohort study of elderly subjects in Italy, it was reported that as adherence to a Mediterranean diet increased, telomerase activity improved, and telomere length increased. The effect of diet on modulation of inflammation and oxidative state stimulates telomerase activity. It is stated that telomerase activity and telomere length are related independently of other variables [87]. Telomeres shorten during cell division and the telomerase enzyme is active in this process to protect telomeres. In a study investigating telomerase activity, it was shown that telomerase activity increased by 29.84% as a result of a comprehensive 3-month lifestyle intervention (a more plant-based diet consisting of low-fat foods and limited refined carbohydrates, physical activity, and stress management) [88].

In some studies, it was found that the relationship between the Mediterranean diet and telomere length differs according to ethnicity and gender [89–92]. Gu et al. [91] study with 1763 individuals aged 65 years and over with different ethnic origins found an association between adherence to the Mediterranean diet and telomere length only in White Americans. In the study of Garcia-Calzon et al. [89], a positive association between adherence to the Mediterranean diet and telomere length was found only in women. Leung et al. [92], in their study on 4758 adult individuals, stated that the Mediterranean diet was positively associated with longer telomeres only in women. In a meta-analysis study showing the effect of gender on telomere length, telomere length was found to be longer in women than in men [90].

Contrary to all these studies, some studies did not clearly find an association between healthy diet models and telomere length [93–95]. In a study of Australian individuals aged between 57 and 68 years, it was observed that there was no any association between diet quality and telomere length [93]. Another study investigated whether there was an association between the DASH diet, The Mediterranean diet, various traditional dietary patterns, and telomere length in Chinese individuals aged 65 years or older. Dietary patterns have been shown to have a minimal role on telomere length [95]. Similarly, a 10-year follow-up study of elderly Finnish men and women showed that the Baltic Sea Diet score, Modified Mediterranean Diet Score, and Dietary Inflammatory Index had little effect on telomere attrition and telomere length. One of the reasons why this study differs from other studies that found an association between telomere length and dietary patterns was explained by the different clinical characteristics of the study population. It is also stated that not all dietary indices may be appropriate for all populations because the content of diet patterns consumed by societies may differ [94].

### The Effect of Specific Foods on Telomere Length

There are scientific studies in which the dietary components in the diet pattern are also associated with telomere length.

#### Foods that Maintain Telomere Integrity

In a 10-year follow-up study investigating the relationship between dietary patterns and telomere length, consumption of nuts, legumes, fruit, seaweed, dairy products, and coffee was positively associated with telomere length [77]. In another study supporting this study, the consumption of legumes, nuts, and fish has been shown to be effective in maintaining telomere length [96]. In addition, some of the dietary components, fruit, vegetable consumption [97, 98], fiber intake [99], and antioxidant nutrients intake [100] are associated with longer telomeres. In a study on rats, it was stated that the telomere shortening increased with red meat consumption in the colon can be reduced by adding resistant starch to the diet. The possible mechanism of this situation is explained as follows. It is stated that the type of protein with increased consumption may cause an increase in the levels of potentially genotoxic protein fermentation products (NH4 + , phenols, and cresols) in the large intestine.

By fermenting resistant starch in the large intestine, fermentation products, especially short-chain fatty acids, are released. The decrease in ambient pH is thought to limit the absorption of potentially toxic biogenic agents [101]. In a study of 5674 people, dietary fiber intake was positively associated with telomere length. It has been reported that an increase of 10 g fiber per 1000 kcal creates a biological aging difference of 5.4 years [102]. In a study by NHANES, it was reported that telomere structure was preserved with increased consumption of nuts and seeds. In the regression model estimates made in this study, it is stated that individuals can experience biological aging in approximately 2 years less if they consume 30 g of nuts or seeds per day [103]. In addition, nuts and seeds consumption is negatively associated with inflammatory markers (C-reactive protein, interleukin-6, fibrinogen) [104]. In this context, nuts and seeds are included in healthy diet models. The healthy nutrition recommendations in the Dietary Guidelines include the consumption of unsalted nuts and seeds in appropriate amounts [105, 106].

Coffee is an important source of antioxidants, along with polyphenols, caffeine, and other bioactive components [107]. Moderate coffee consumption is inversely related to inflammatory markers [108]. Coffee consumption and caffeine intake levels differ between individuals. In a study investigating the effect of this difference, coffee consumption was positively associated with telomere length. It has been reported that the effect of coffee on telomere length may be related to the caffeine and other bioactive components in its content [109]. In a study of the NHANES conducted with 5826 adults, coffee consumption and caffeine intake levels were assessed from a 24-h dietary recall. In the statistical analysis by controlling for covariates, it was reported that for every 100 g of coffee consumed, telomeres are on average 15.0 base pairs longer (*F* = 12.6, *p* = 0.0013). It has been suggested that coffee consumption may slow aging through its positive association with telomere length. However, in the same study, it was reported that caffeine intake was inversely related to telomere length (*F* = 15.1, *p* = 0.0005). In this study, which evaluates coffee consumption and caffeine intake separately, it is shown that other components other than caffeine are responsible for the protective effect of coffee on health. In addition, the inverse relationship between caffeine and telomere length is also noted to be consistent with dose–response [110]. In addition to all these studies, it was noted that tea, which is another source of caffeine and polyphenols other than coffee, could protect telomeres from oxidative damage with its antioxidant properties. In Chinese men, tea consumption was found to be positively associated with telomere length [111]. As yet, the effect of caffeine on the maintenance of DNA integrity has not been clarified. In a biological study, it is stated that caffeine prevents oxidative DNA breakage, while caffeine may also have a pro-oxidant effect [112]. In a study using yeast as a model organism, caffeine was found to shorten telomeres [113]. There are limited research results examining the effects of coffee and caffeine on telomere length, which are not yet clear, and cross-sectional designs that make direct causal inferences difficult. Considering the dose–response consistency in the undesirable effect of caffeine on telomere length, it may be useful to keep in mind the recommendations of international organizations regarding caffeine consumption. In the report published by the European Food Safety Authority (EFSA), it has been reported that a single dose of caffeine intake of up to 200 mg for adults is safe, and additionally, 400 mg of caffeine (about 4 cups of coffee) intake during the day will not cause health problems. In the same report, the safe caffeine intake dose for pregnant and lactating women is 200 mg [114]. In the World Health Organization (WHO) report, it is recommended to limit pregnant women who consume more than 300 mg of caffeine due to possible health risks [115].

In a study of NHANES conducted on 5834 adults, the effects of cow’s milk consumption and milk fat on cellular aging related to telomere length were examined. When adjusting for all the covariates, adults consuming skim or 1% milk were found to have significantly longer telomeres than adults consuming full-fat or 2% milk. Additionally, those who consume skim milk have been shown to have telomeres approximately 115 base pairs longer than those who do not drink cow’s milk [116]. The results of this study are in line with the data of the Dietary Guidelines for Americans (2015), which recommends low-fat and skim milk consumption for adults [105]. Similarly, another widely accepted healthy eating model (the healthy plate model) supports this study with a recommendation for milk consumption [117]. The portion amount recommended for adults in Turkey Dietary Guideline is similar to these [106]. Lee et al. [77] stated that although dairy products consumption has a positive effect on telomere length, saturated fat intake may increase with increasing milk consumption, and this may negatively affect telomere length. Due to the saturated fat and cholesterol content of dairy products, they are recommended to be consumed in recommended amounts and for individuals in risk groups, fat-free or low-fat consumption is recommended [105].

#### Foods that Increase Telomere Shortening

Some studies draw attention to dietary components that are negatively associated with telomere length and that are thought to accelerate aging by affecting the shortening of telomeres. The foods and nutritional components positively associated with telomere shortening in these studies are white bread, sugar-sweetened beverages, processed meat, butter, total fat intake, saturated fat intake, and increased alcohol consumption.

In the study of Garcia-Calzon et al. [100], it has been reported that a daily increase of one serving (60 g) of white bread consumption increases the risk of low telomere length by 37%. It has been stated that increased consumption of white bread, which has a high glycemic load, increases oxidative stress and thus accelerates inflammation-induced telomere shortening. It is stated that sugar-sweetened beverages which are consumed regularly in the daily diet may affect the development of diseases by accelerating cell aging [118]. There is another study supporting this by showing that sugar-sweetened beverages are a risk factor for shortening telomeres (*β* =  − 0.120, *p* = 0.004) [96].

An in vitro study investigated the association of pro-inflammatory conditioning and high glucose intake with telomere shortening. Pro-inflammatory conditioning increased telomere shortening. However, higher glucose intake alone was not associated with faster telomere shortening [119]. In a 10-year follow-up study, which had a higher level of evidence than the in vitro study, the consumption of red meat, processed meat, and sugar-sweetened soda was associated with shorter telomere length [77].

Tiainen et al. [97] found that men consuming high amounts of butter had significantly shorter telomeres than men consuming low amounts of butter (*p* = 0.05). Total fat intake and saturated fat intake (respectively *p* = 0.04 and 0.01) have been shown to be inversely related to telomere length.

An inverse relationship between processed meat consumption and telomere length has been shown in a cross-sectional study of adults of different ethnicities. It has been determined that individuals, who consume one serving or more of processed meat per week, have a shorter telomere length than individuals who do not [120]. One of the previous studies emphasized that the negative effect of processed meats on telomeres can induce inflammatory mediators due to their high fat and protein content [121]. In a cross-sectional study of American Indians, the association between meat consumption and telomere length differs depending on whether the meat was processed. It was found that for each one serving of processed meat consumed daily, telomere length was 0.021 units shorter (β ± SE =  − 0.021 ± 0.008, *p* = 0.009), and there was no association between unprocessed meat consumption and telomere length [122].

In addition to these studies showing a negative relationship between processed meat and telomere length, in the report published by the World Health Organization, it is recommended to limit the consumption of processed meat as much as possible to prevent diseases such as cancer [123].

Although red wine from fermented beverages has been shown to have a positive effect on telomere length in the Mediterranean diet, there is also a study showing that long-term moderate alcohol consumption is associated with lower telomere length. In this study, the clear mechanism explaining the relationship between alcohol consumption and telomere shortening could not be revealed by the researchers. However, it is stated that this observed negative effect may be related to increased oxidative stress, impaired antioxidant function, or disruptions in telomerase activity [124]. Another study supporting this study, increased alcohol consumption and intake of short and medium-chain saturated fatty acids were negatively associated with telomere length in post-menopausal women [125].

### The Effect of Dietary Antioxidant Intake on Telomere Length and Aging

Considering that oxidative stress and inflammation may adversely affect telomere length, increased antioxidant intake with nutrition may have a protective effect on telomere length. In this context, when the studies are examined, positive effects are seen.

One of the most important health benefits of phenolic compounds, which are bioactive plant components found in whole grain products, is that they act as antioxidants by giving hydrogen atoms to free radicals [126]. In a study conducted on a population of middle and elderly women, telomere length was positively associated with dietary fiber intake, while waist circumference and linoleic acid intake were negatively associated with telomere length. In addition, it was noted in the study that telomere length may be affected through anti-inflammatory and antioxidant mechanisms [99]. In a cross-sectional study of Brazilian children and adolescents aged 7–17 years, individuals who regularly ate fruits and vegetables were found to have longer telomeres. It is noted that antioxidants from vegetables and fruits are associated with the maintenance of the telomere biology of individuals [127].

In a 10-year follow-up study examining the relationship between micronutrient intakes and telomeres, folate, vitamin C, and potassium intakes were positively associated with telomere length [128]. There are other studies supporting this study. Individual nutrient intakes such as folate, vitamins C, E, D, and A, and carotenoids, magnesium, and omega-3 fatty acids are positively associated with longer telomere length [129–131], and shortening of telomeres can be reduced by omega-3 uptake [125, 130]. In a study conducted with the elderly who are cognitively healthy, the effect of a walnut-added diet, corresponding to 15% of daily energy, on telomere shortening was investigated. It was reported that walnut, a source of omega-3 added to the normal diet, tended to reduce leukocyte telomere attrition compared to the control group after 2 years [132]. In a study conducted by Richards et al. [133] on women, it was found that higher serum vitamin D levels were associated with longer telomeres. The increase in dietary intake of selenium, which is another powerful antioxidant, has also been shown to have a protective effect on telomere length [134].

It is assumed that the oxidative stress load is reduced by the consumption of diets or foods rich in antioxidant nutrients. Plant-based diets may delay telomere length shortening, given that oxidative stress may cause telomere attrition [135].

### The Potential Mechanisms of Healthy and Unhealthy Nutrition on Telomere Length

Telomeres in the nucleoprotein structure, which prevent the degradation of chromosomes, are located at the ends of the chromosomes [136] and gradually shorten with each cell division [137]. In addition to existing mechanisms during cell division, oxidative stress has been shown to cause DNA damage and telomere attrition in a dose-dependent manner [56, 138, 139]. Aviv [140] states that telomere shortening can be observed in conjunction with the harmful effect of hydroxyl radicals, which can cause DNA damage. There are also studies showing that reactive oxygen species (ROS) induce oxidative modification and damage to telomeres [141, 142]. In this context, oxidative stress has a negative effect on telomere erosion [141], and consumption of foods with high antioxidant content has a positive effect on telomere length [130, 131, 135].

Houben et al. [141] emphasized that different pathological conditions modulated by oxidative stress and chronic inflammation affect the rate of shortening of telomeres and thus may affect the human lifespan with aging and chronic disease.

**Fig. 2:**
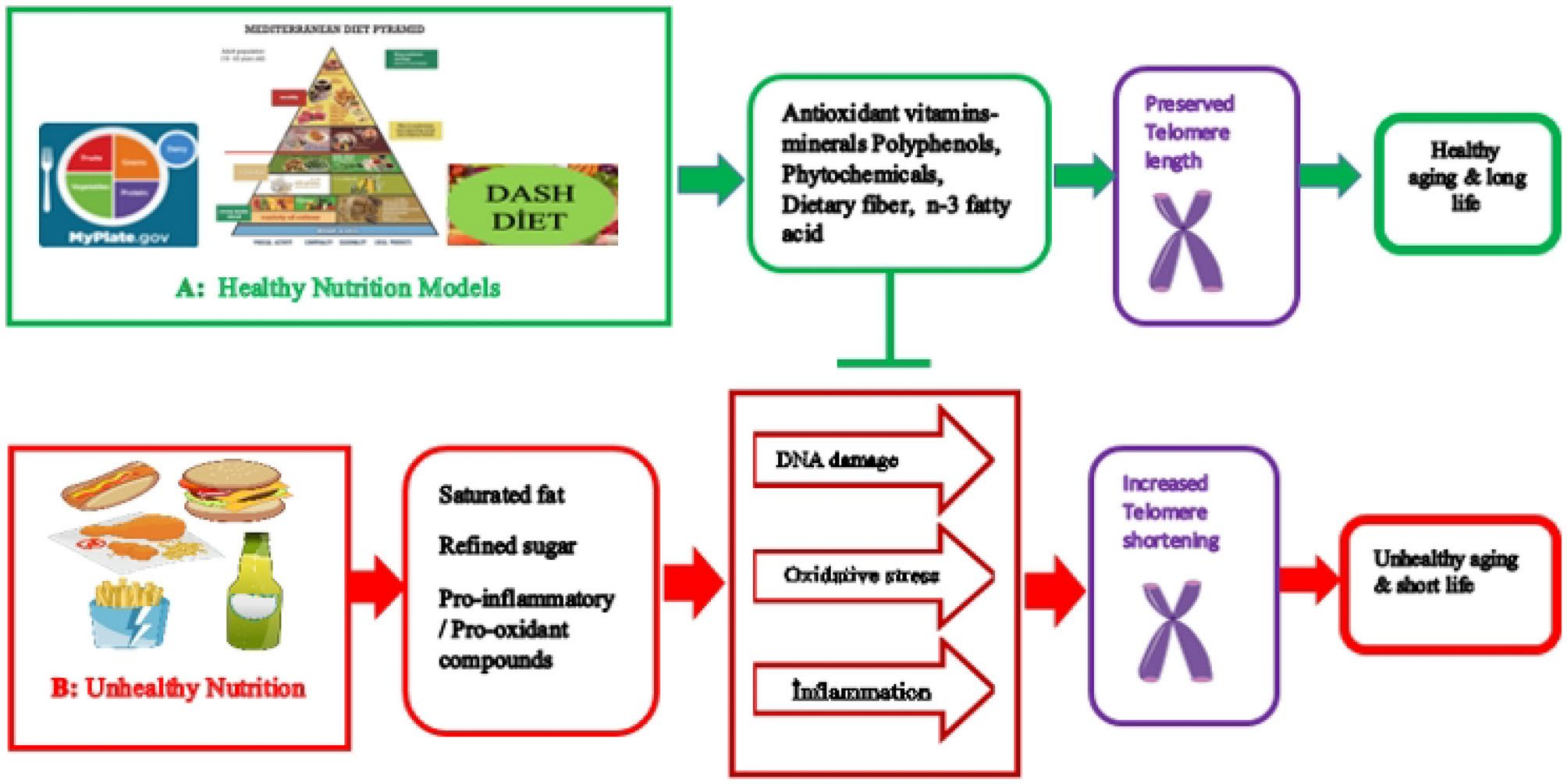
The potential mechanisms of healthy and unhealthy nutrition on telomere length (the Mediterranean diet pyramid in the picture is taken from D’Alessandro et al. [143])

Studies focusing on the relationship of telomere length with nutrition have examined the effects of dietary patterns, some foods, and nutrients. In this context, the effect of nutrition on telomeres can be mentioned in 2 different mechanisms (as A and B pathways) as shown in Fig. 2.

In A pathways, it should be emphasized that individuals who eat healthy diets with high antioxidant content [78, 79, 117] recommended by international organizations can protect their DNA integrity. Thus, regardless of chronological age, a healthy life can be led by contributing to the preservation of telomere integrity with a significant decrease in telomere shortening rate (Fig. 2A) [82, 86].

In B pathways, some dietary patterns and foods (Western-style diet, refined carbohydrates, processed meat, saturated fat, etc.) are associated with insulin resistance, chronic inflammation, and oxidative stress. These effects of unhealthy foods are associated with high amounts of refined sugar, saturated fat, and low amounts of antioxidant vitamins, minerals, and fiber. Unhealthy cooking methods applied to foods can also cause the accumulation of pro-inflammatory and pro-oxidative compounds in foods. Due to all these factors, increasing telomere shortening may accelerate the aging process and increase age-related chronic diseases (Fig. 2B) [120–122, 132].

Most of the studies investigating the association between nutrition and telomeres used FFQ and 24-h dietary recall. More clinical and longitudinal studies are needed to obtain stronger evidence and establish a causal relationship.

## Conclusion, Limitation, and Future Research

We suggest that ensuring energy balance with regular physical activity and healthy diets can contribute to the aging process by protecting telomere length. However, there are some limitations to our study. Firstly, different methods in studies, short and inconsistent durations, different types of exercise, different diet patterns, and non-standard foods have led to conflicting results. Secondly, it should be acknowledged that the utilization of telomere length as an aging metric might encounter limitations due to the heterogeneous cellular origins of the samples under investigation. While telomere length is predominantly assessed in telomeric DNA regions, it is important to recognize that certain studies, including some cited in the article, have employed diverse cell types such as whole blood or saliva cells for this purpose. This variance in cellular composition could contribute to the observed discrepancies in results across studies. Another critical aspect to consider pertains to the methodologies employed for the quantification of telomere length. The accuracy and precision of the measurement techniques can substantially influence the obtained outcomes. It is imperative to acknowledge that studies evaluating telomere length have employed a range of methodologies, leading to potential variations in the precision of the measurements [144, 145]. This variation in measurement precision could potentially contribute to the incongruent findings observed in the literature. In addition, studies on basal metabolic rate and resting metabolic rate, important factors of energy balance, were not found. These limitations underscore the complexity of utilizing telomere length as a consistent measure of aging. Acknowledging the diversity in cell types and the intricacies of measurement methodologies is pivotal in interpreting and reconciling the sometimes contradictory findings within the existing body of research.

In future studies, answers can be sought to the questions of “Do basal metabolic rate and resting metabolic rate affect the aging process through telomere protection? “How does the combination of a healthy diet pattern and long-term exercise affect telomere length?” In addition, more studies are needed to elucidate molecular-based mechanisms.
